# Nontargeted Effect after Radiotherapy in a Patient with Non-Small Cell Lung Cancer and Bullous Pemphigoid

**DOI:** 10.1155/2015/964687

**Published:** 2015-08-09

**Authors:** Carsten Nieder, Khalid Al-Shibli, Terje Tollåli

**Affiliations:** ^1^Department of Oncology and Palliative Medicine, Nordland Hospital, 8092 Bodø, Norway; ^2^Department of Clinical Medicine, Faculty of Health Sciences, University of Tromsø, 9038 Tromsø, Norway; ^3^Department of Pathology, Nordland Hospital, 8092 Bodø, Norway; ^4^Department of Pulmonology, Nordland Hospital, 8092 Bodø, Norway

## Abstract

*Purpose*. To describe tumor shrinkage of nonirradiated lung metastases in a patient with non-small cell lung cancer. *Case Report*. The patient had a concurrent autoimmune condition, bullous pemphigoid, which was clinically exacerbated during radiotherapy of mediastinal and axillary lymph node metastases. He also developed a series of infections during and after irradiation, and we hypothesize that the immunological events during this phase might have influenced the size of the nonirradiated metastases. *Conclusion*. Ionizing radiation generates inflammatory signals and, in principle, could provide both tumor-specific antigens from dying cells and maturation stimuli that are necessary for dendritic cells' activation of tumor-specific T cells. Even if the detailed mechanisms causing nontargeted immune modulatory effects in individual patients are poorly understood, clinical development of radioimmunotherapy is underway.

## 1. Introduction

It has been long realized that ionizing radiation is able to influence cells and tissues that are not directly located in the radiation fields [1–3]. Several case reports, experimental studies, and reviews have addressed such phenomena, which typically are termed bystander, nontargeted immune modulatory, or abscopal effect. In clinical practice, few patients experience tumor response in metastatic sites that are distant from the radiation fields. Here we describe the clinical course of a patient with nonsmall cell lung cancer (NSCLC) and bullous pemphigoid, an autoimmune skin disease that typically manifests in older people and with male preponderance, without clear association to malignant diseases [4], who developed response of nonirradiated lung metastases, possibly related to immune activation by infection.

## 2. Case Report

**Figure 1:**
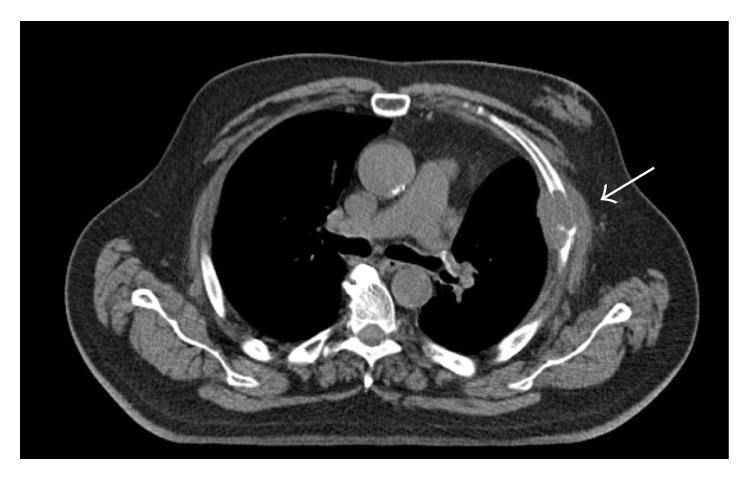
Computed tomography scan of the thorax showing left-sided local relapse in the pleura and thoracic wall after lobectomy. Status before palliative radiotherapy.

**Figure 2:**
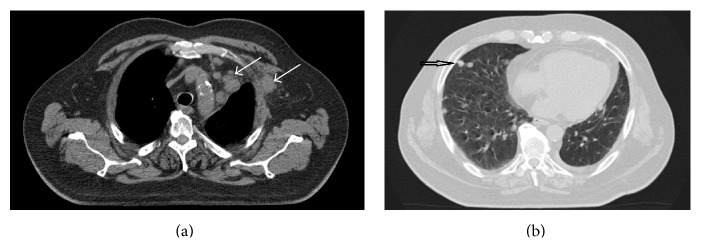
(a) Computed tomography scan of the thorax showing left-sided axillary nodal metastasis and mediastinal lymph node metastases. Status before palliative radiotherapy. (b) Computed tomography scan of the thorax showing right-sided pulmonary metastases (example of the patient's bilateral metastases). During radiotherapy, this region received a cumulative dose of <1 Gy.

**Figure 3:**
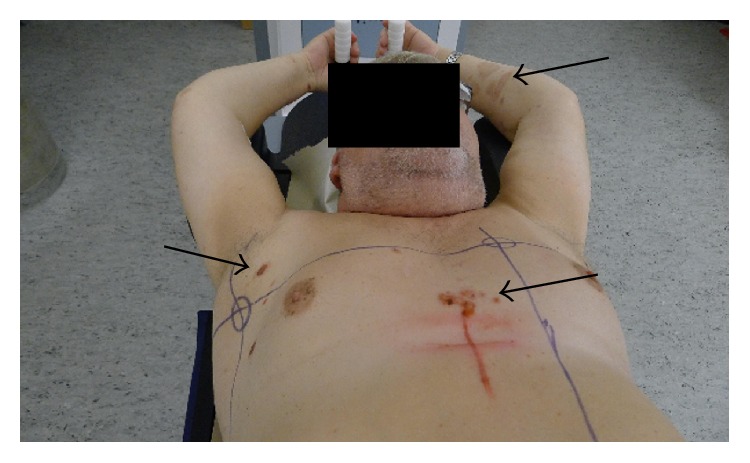
Skin lesions at the time of simulation before palliative radiotherapy to the mediastinum and left axilla. Bullous pemphigoid mainly involving trunk and arms.

**Figure 4:**
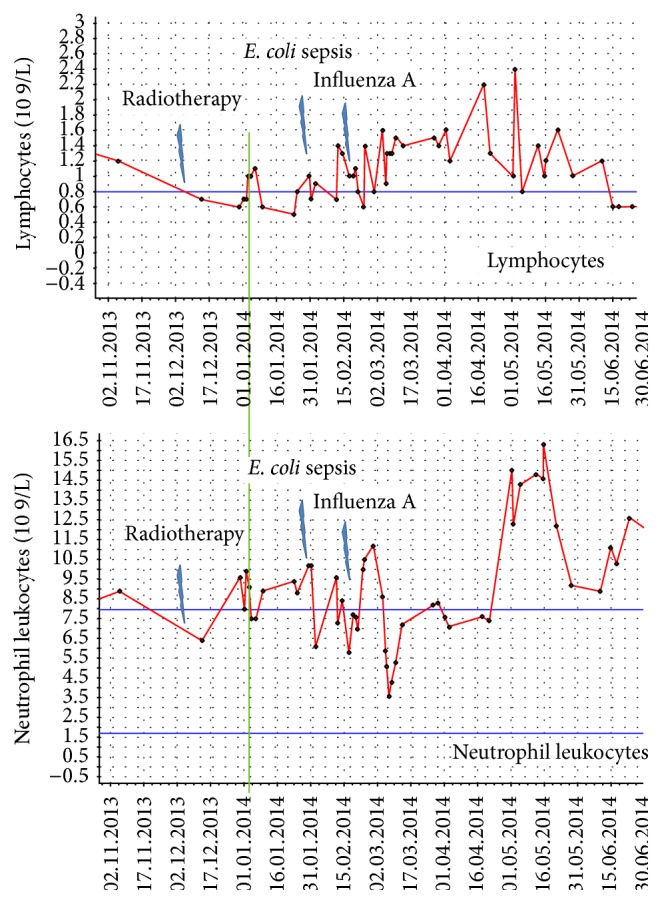
Time course of neutrophil leukocyte and lymphocyte counts during follow-up. The green line corresponds to the date of the computed tomography scan showing the nontargeted effect. At this time, the patient had recovered from presumably radiation-related lymphopenia. Abnormally high lymphocyte counts were never detected. In contrast, several episodes of leukocytosis occurred.

**Figure 5:**
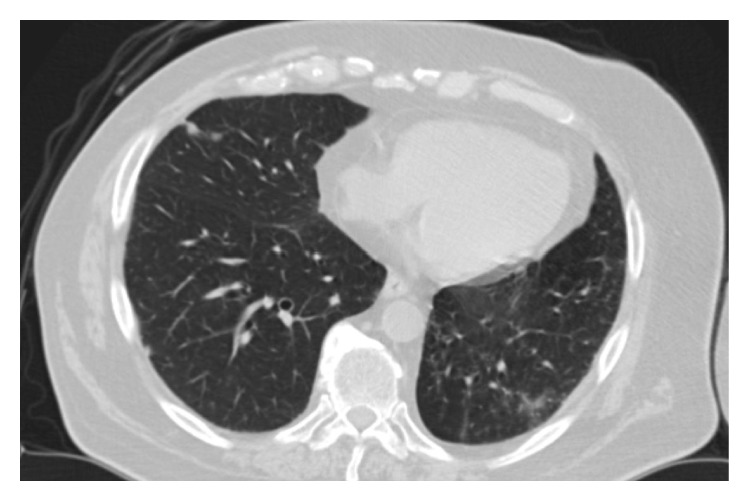
Computed tomography scan of the thorax showing right-sided pulmonary metastases approximately 3 weeks after radiotherapy to other targets. Marked reduction in size.

**Figure 6:**
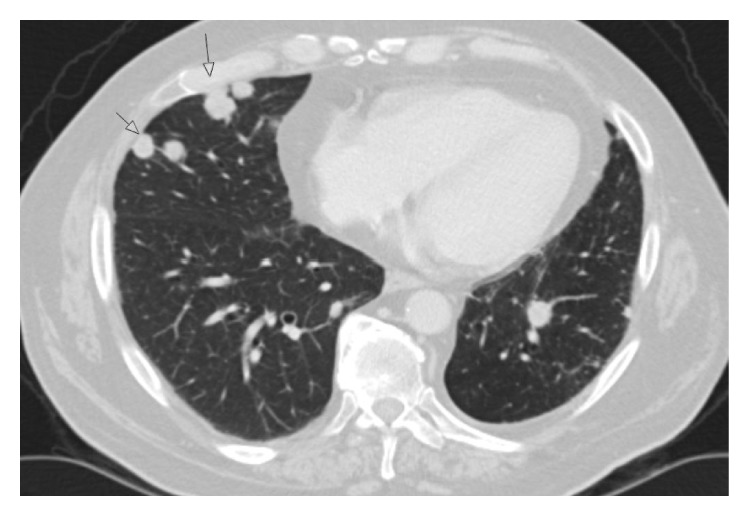
Computed tomography scan of the thorax showing progression of the right-sided pulmonary metastases approximately 3 months after radiotherapy to other targets. Temporary nontargeted effect.

A 67-year-old Caucasian male patient was treated for NSCLC of the left upper lobe in February 2013 (lobectomy). He had a history of heavy smoking, chronic kidney failure, and myocardial infarction. Furthermore, he had been treated with radical prostatectomy (2009) and salvage radiotherapy to the prostate bed (2010, stage pT4 N0 R1, Gleason score 4 + 4). Because of new biochemical relapse, he had started antiandrogen treatment with bicalutamide in November 2011. His prostate-specific antigen (PSA) value was <0.2 ng/mL after endocrine therapy. Cystectomy had been performed in March 2012 after diagnosis of WHO grade 3 urothelial cell bladder cancer (stage pT1). Medical treatment also included simvastatin 40 mg daily, acetylsalicylic acid 75 mg daily, cetirizine 5 mg daily, hydrochlorothiazide 25 mg daily, and valsartan 320 mg daily. Histology after lobectomy showed two foci of adenosquamous carcinoma (22 and 23 mm, resp.) invading the visceral pleura (stage pT3 pN1). Immunohistochemistry of the two tumors showed positive staining for p53 in only one of them. Adjuvant chemotherapy started in April 2013. Because of impaired renal function, carboplatin AUC6 and vinorelbine 30 mg/m^2^ were prescribed. After three of four planned cycles, the patient developed local relapse in the thoracic wall and skin changes, mainly at the upper part of the body and arms, which were diagnosed as bullous pemphigoid. White blood cell count and differential were within the normal range. Treatment included prednisolone and topical desoxymethasone. In July 2013, 3D conformal palliative radiotherapy was administered to the region of pleural/thoracic wall recurrence (13 fractions of 3 Gy), Figure 1. In November 2013, systemic progression was detected, with new bilateral pulmonary metastases and mediastinal and left-sided axillary lymph node metastases, Figures 2(a) and 2(b). A new course of 3D conformal palliative radiotherapy was given (10 fractions of 3 Gy to all lymph node metastases). During radiotherapy, the bullous pemphigoid was exacerbated and secondary infection occurred (status at simulation shown in Figure 3). Serum C-reactive protein was elevated to 50 mg/L and leukocyte count was slightly elevated to 11.1 × 10^9^/L (Figure 4). The daily dose of prednisolone during radiotherapy was 20 mg. The patient received antibiotic treatment with cefuroxime. Two weeks after the last fraction of radiotherapy, the patient was hospitalized and treated for pneumonia. Chest X-rays showed a pneumonic infiltrate in the left lower lobe. Microbiological tests did not reveal the cause of infection. Three weeks after discharge, he was hospitalized again, this time because of sepsis caused by* E. coli*. Computed tomography (CT) showed partial response of the irradiated and nonirradiated lesions (Figure 5, January 2014). The total radiation dose in the region of these lung metastases was <1 Gy, compared to the prescription dose of 30 Gy. Two weeks after discharge, he was treated for another infection, now caused by influenza A virus. In April 2014, CT showed progression of all pulmonary metastases (Figure 6). Further systemic therapy was not given because of reduced performance status, impaired kidney function, and frequent infections. The patient received analgetics and supportive treatment under guidance of the hospital's multidisciplinary palliative team. The bullous pemphigoid never disappeared completely. He died from pulmonary failure in July 2014, seventeen months after surgical resection of the primary tumor.

## 3. Discussion

NSCLC is often characterized by rapid development of distant metastases, even in patients receiving radical treatment upfront [5]. Palliative radiotherapy is an established and effective option in this setting [6, 7]. The patient described here experienced lasting local control of all irradiated tumors. Surprisingly, his unirradiated lung metastases also responded rapidly, followed by progression later on. This so-called nontargeted immune modulatory effect is a rare event. Previous case reports often related distant responses outside of the radiation fields to administration of immune modulating agents [8]. Immunogenic forms of tumor cell death induced by X-rays might include immune modulating danger signals like heat shock protein 70, adenosine triphosphate, and high-mobility group box 1 protein [9]. Moreover, antitumor effects exerted by cells of the innate (natural killer cells) as well as adaptive immune system (T cells activated by dendritic cells) might play a role. Ionizing radiation generates inflammatory signals and, in principle, could provide both tumor-specific antigens from dying cells and maturation stimuli that are necessary for dendritic cells' activation of tumor-specific T cells. Experimental data provided support for these mechanisms [1]. The patient described in our report had a concurrent autoimmune condition, bullous pemphigoid, which was clinically exacerbated during radiotherapy. He also developed a series of infections during and after irradiation, and we hypothesize that the complex immunological events during this phase might have influenced the shrinkage of the nonirradiated metastases. Unfortunately, his immunological status was not analyzed in detail. Future studies should include longitudinal analyses of different immune cell populations, ideally also tumor-infiltrating lymphocytes. Current research efforts aim at development of clinically applicable protocols of combined radio- and immunotherapy [10].
